# Genome Sequence of Naphthalene-Degrading Soil Bacterium *Pseudomonas putida* CSV86

**DOI:** 10.1128/genomeA.00234-12

**Published:** 2013-02-21

**Authors:** Prashant S. Phale, Vasundhara Paliwal, Sajan C. Raju, Arnab Modak, Hemant J. Purohit

**Affiliations:** a Department of Biosciences and Bioengineering, Indian Institute of Technology-Bombay, Powai, Mumbai, India; b Environmental Genomics Division, CSIR-National Environmental Engineering Research Institute, Nagpur, India; c MEM-Group, Department of Biosciences, University of Helsinki, Helsinki, Finland

## Abstract

*Pseudomonas putida* CSV86, a soil isolate, preferentially utilizes naphthalene over glucose as a source of carbon and energy. We present the draft genome sequence, which is 6.4 Mb in size; analysis suggests the chromosomal localization of genes coding for naphthalene utilization. The operons coding for glucose and other aromatic compounds might also be annotated in another study.

## GENOME ANNOUNCEMENT

*Pseudomonas* is found to be present in a wide range of ecological niches, with metabolic versatility and an ability to adapt to different environmental conditions. This is reflected in its large genome size, with unique gene organizations. The genome sequencing and its analysis facilitate the understanding of the multiplication of *Pseudomonas* and its contribution to different aspects, such as bioremediation, biocontrol, and pathogenicity (1). Despite its metabolic versatility, the wild-type strains of *Pseudomonas* do not perform efficiently in the bioremediation process. This is due to the availability of simple carbon sources, like sugars and organic acids, in the environment. Attempts have been made to engineer organisms for the efficient utilization of aromatics in the presence of glucose. However, the stability and viability of such strains in nature pose a challenge (2). 

A soil isolate, *Pseudomonas putida* CSV86 (herein referred to as CSV86), utilizes naphthalene, methylnaphthalenes, phenylacetic acid, 4-hydroxyphenylacetic acid, salicylate, benzyl alcohol, benzoate, and *p-*hydroxybenzoate as sole sources of carbon and energy (2–10). CSV86 lacks plasmids, and based on preliminary studies, it was hypothesized that the naphthalene-degradation genes are located on the host chromosome, probably as a genomic island (6). CSV86 has been found to have a novel property of preferential utilization of aromatics over glucose, and it cometabolizes aromatics and organic acids (2, 4, 5, 9, 10). This makes CSV86 an ideal candidate for studying carbon catabolite repression and to engineer new strains for effective bioremediation.

To understand the organization of aromatic catabolic pathways, the genome of strain CSV86 was sequenced using Roche 454 GS (FLX Titanium) platform. The high-quality reads of 867,565 bp were assembled with Newbler v2.0, a 454 assembly tool, into 228 contigs with a sequencing coverage of 61.08-fold, with an average read length of 428 bp. The 6,472,491-bp draft genome has an average G+C content of 61.85%. The genome was annotated using Rapid Annotations using Subsystems Technology (RAST) v4.0 (11) and the NCBI Prokaryotic Genomes Automatic Annotation Pipeline (PGAAP) (http://www.ncbi.nlm.nih.gov/genomes/static/Pipeline.html). NCBI PGAAP annotated 209 high-quality contigs into 5,900 genes, 5,836 coding sequences (CDSs), 60 tRNA genes, 3 copies of 5S rRNA genes, and 1 copy of a 16S rRNA gene. Nine mobile elements were identified by the Insertion Sequence (IS) Finder database (http://www-is.biotoul.fr//) with zero *E* value as the threshold (12); the Tandem Repeats Finder program predicted 173 tandem repeats in the genome (13). 

The upper pathway genes of naphthalene degradation in CSV86 were identified and found to be located next to the genes encoding tRNA-Gly and integrase. This supports earlier observation that the naphthalene-degradation genes are part of a genomic island (6). Similarly, the genes encoding enzymes involved in the degradation of salicylate, benzoate, 4-hydroxybenzoate, phenylacetic acid, hydroxyphenyl acetic acid, and homogentisate pathways were also identified. An operon encoding various components of the glucose transport in CSV86 was also identified. Various genes and gene clusters responsible for the heavy metal resistance were also identified.

### Nucleotide sequence accession numbers. 

This Whole Genome Shotgun project has been deposited at DDBJ/EMBL/GenBank under the accession no. AMWJ00000000. The version described in this paper is the first version, AMWJ01000000.
